# Sanitary Reform in Texas

**Published:** 1899-09

**Authors:** 


### SANITARY REFORM IN TEXAS.

### Two Prize Papers on Sanitation.

[As stated in the JOURNAL last month, the medical profession and the municipal authorities of San Antonio have inaugurated a sanitary reform in that famous city, and are pushing it with most commendable vigor. The movement is backed by the people, and to stimulate additional interest in the subject cash prizes made up by subscription on the part of prominent business men were offered for the best paper on, respectively, "City Sanitation" and "Home Sanitation;" the first to be competed for solely by non-professionals, and the latter solely by women. The first prize for the best paper on "City Sanitation" was awarded to Mr. Jno. Rodham, of San Antonio, and first prize for paper on "Home Sanitation" was given to Mrs. A. Fletcher, also of San Antonio.]

"CITY SANITATION.

"BY JNO. RODHAM, SAN ANTONIO, TEXAS.

"In discussing the subject of 'City Sanitation' the limited length of the article prevents one from going into details. I will content myself with making such suggestions as occur to me for the improvement of the sanitary condition of the city.

"*Water.*—The first essential of a modern city is an adequate supply of pure water at low cost. We have this in San Antonio, but unfortunately there is a considerable class of citizens who are unable to pay the tariff demanded by the waterworks company and who reside upon the Alazan creek and the ditches and drink the water from such polluted sources. It is from such people that epidemics usually arise, and measures should be taken at once to supply these people with pure water by means of free hydrants, without charge, and to prevent them from using the infected water of ditches and streams.

"*Public Baths and Wash Houses.*—Another essential of a city such as this, situated in a warm climate, is that abundant opportunities should be afforded to the people, of the lower classes in particular, for frequent bathing and for washing their clothing. Those of us who take a bath every day cannot appreciate the condition of many of the poor who have no bath tubs and do not take a bath once in a month. Now that the flow of the river has diminished, and it is no longer fit to bathe in, the city should establish a number of public baths and wash houses at different points where poor people can bathe for nothing, or for a nominal price. The wash houses should be provided with boilers, hot water, mangles and the other labor-saving machinery usually installed in such places, and persons should be permitted for a nominal consideration to use them and wash their clothing there. Such places are in successful operation in Paris, France, and Manchester, England, and numerous other places. The buildings can be erected and equipped at small cost. Experience has shown that wells bored to a considerable depth near to the river bed always produce a large and permanent supply of artesian water. Let several of them be dug at different points in the city and the bath houses be erected near to them. The water necessary can in this manner be obtained at small cost.

"*Public Drinking Fountains.*—It is very surprising to a stranger in the city to notice that, in spite of the hot weather usually prevailing, there is not one public drinking fountain in the city. It is an extraordinary fact that in this city of 50,000 you cannot get a drink of water without asking at some saloon or store for it, with the slight exception of some of the hydrants in the court house. This should not be. Water is essential to health, and plenty of it for drinking purposes should be free to all and easily accessible. A number of fountains should be established at public places on the plazas and streets without delay.

"*Sewerage.*—We are blessed with an almost perfect system of sewerage. It will compare favorably with that of any other city. The difficulty of disposing of the sewerage can be remedied in the easiest manner by extending an irrigating ditch in a southerly direction from the outfall sewer, and leasing the water for irrigation purposes to adjoining landholders.

"*Public Closets and Urinals.*—As an incident to a proper sewerage system there should be established a system of public urinals and water closets. In a city having any pretensions to civilization it is barbarous and extraordinary that citizens and strangers in the city should be required to depend upon the charity or courtesy of others for a place in which to relieve the calls of nature. It tends to indeceny as well, and results in the nostrils of pedestrians in our by-streets by night being assailed with a large variety of noxious and unpleasant odors. Much injury to the health of the young and delicate results from being prevented from relieving themselves at the call of nature by reason of the absence of these facilities. A number of these urinals and water closets should be established at convenient points.

"*Sewer Connections and Cleaning of Lots.*—The most difficult problem that the city has to deal with is the enforcement of the sanitary regulations governing homesteads and their surroundings. This arises from our homestead law, which prevents the city from taxing the cost of sewer connections, or of cleaning up the same, or any sanitary improvement, or penalty, against the property. The present laws in regard to the enforcement of the cleanliness of yards and dwellings and of compelling the making of sewer connections are good if they could be enforced. No tax or assessment can be levied against the homestead for the enforcement of these ordinances, and if the city does the work she will generally be unable to collect the cost from the owner. An energetic and continuous enforcement of the criminal ordinances would, in my opinion, result in compelling most of the offenders to comply with the law.

"I would suggest that the following changes in the ordinances of the city might be useful to that end. In the first instance to be mentioned, the city's charter would have to be amended to give the requisite authority, and possibly in the second instance.

"First.—The city should have the right to condemn property under proper condemnation proceedings for public use when the condition of the property is such (by reason of non-compliance with the sanitary ordinances) that it is a public nuisance or a menace to the public health.

"Second.—The city shall have the right, where a person fails to keep his premises clean, or to make the proper sewer connection, to clean up said premises and make said connections, and that upon proper complaint and proof that the owner has failed to comply with the ordinance and the cost of making the necessary connection, etc., that the amount of such cost, together with a suitable penalty, shall be charged against him, and the offender shall be remanded to jail until paid. That is to say, that the amount of the penalty against him should be the cost of the work and something extra.

"There is no good reason why the city prisoners should not be employed to clean up premises where owners have failed to do so. In this way it might occur that the owner of property would be fined for failing to clean up his premises, and by an act of poetical justice be set to work with the chain gang to clean up his own lot. A few examples of this kind would be wholesome object lessons.

"The condemnation of private property, where the same has become a menace to public health, has been successfully carried out in Birmingham under the administration of the Hon. Joseph Chamberlain, when he won his first political spurs as mayor of that town. This need only be resorted to in extreme cases, but the exercise of this right should be insisted upon by citizens whenever the case demanded it. Many places which can never be cleaned or purified except by being pulled down and rebuilt can in this way be rendered clean and sanitary. Of course, the owners would be paid the fair value of the property.

"*Pure Food.*—Infant mortality very largely results from impure or infected milk. An inspector should be appointed, or a number of them, whose duty it should be to inspect samples of milk sold to the inhabitants, and to see to it that it is of the required standard of purity, not watered, and free from tubercle and other germs. The same system should be carried out in regard to the more important articles of food, and particularly in regard to meat, canned goods, sausage, bacon, etc. A proper inspection of food and the prompt destruction of that which is unfit for consumption would save many lives annually.

"*Trash Cans and Garbage Crematory.*—The crematory owned by the city should be utilized to burn the garbage of the city, as well as the dead animals, instead of dumping it outside the city limits. A uniform system of trash cans should be enforced, and the trash carts should make their rounds more extensive and make them oftener and earlier in the morning.

"*Street Sprinkling.*—Dust is a serious injury to health. The principal streets that are not paved should be sprinkled with crude oil or oil waste. This system has been tried in Fort Worth and also upon the Pennsylvania Railroad and is much cheaper and more effective than water. One sprinkling allays the dust absolutely, and does not have to be repeated for a very long time.

"*Expectoration Upon Sidewalks.*—It is disgraceful that many men are allowed to defile the sidewalks with large splatches of tobacco juice and old tobacco quids. This is sickening to the sight, and the effect upon the dresses of ladies which trail along the sidewalks is disastrous. An ordinance should be passed and enforced to prevent expectorating upon the sidewalks. An ordinance should also be passed prohibiting consumptives from expectorating in the streets anywhere. The infectious nature of the sputum of consumptives is well known. When upon the streets, they should be required to expectorate either in a handkerchief or in a small cardboard box or other receptacle carried by them, and such handkerchief or other receptacle should be subsequently destroyed by fire.

"*Street Sweeping.*—The paved streets should be regularly swept, and the ordinance in regard to sweeping sidewalks should be rigidly enforced. Ornamental trash cans should be placed at the corner of every block. Citizens should be prohibited from throwing banana peels, waste paper and other trash upon the streets and required to place them in such trash boxes. By this, I refer to pedestrians particularly. Street sweepers should be stationed on the paved streets in the day time, each with a section of the street in his charge, and it should be their duty to keep their section clean during the day and remove therefrom all trash, waste, horse droppings, etc., and deposit the same in said trash cans.

"*Disinfection.*—A Formaldehyde disinfecting apparatus should be obtained by the city and the premises where persons have died of infectious diseases should be disinfected thereby. Consumptives should be moved to a separate ward of the hospital, and if they remain at home the relatives and attendants should be instructed in the simple methods of avoiding infection, and directed to destroy the sputum of the patient. In case of death the bedding, etc., should be destroyed, and the house renovated and floors washed with antiseptics.

"*Societies.*—Clubs should be formed in every ward and section of the city to induce and enforce cleanliness and sanitation. Lectures on the subject should be given in the schools. Prizes should be given, either by the city or such clubs, to the school children for the best kept yard and sidewalk in each ward. As the duty of cleaning the yard, and often the sidewalk, usually devolves upon the juvenile members of a family, the offering of prizes would cause a great amount of emulation among them and result in a great step toward cleanliness."

---

### HOME SANITATION.

BY MRS. A. FLETCHER, SAN ANTONIO, TEXAS.

"Four essentials to good health are cleanliness, sunlight, pure air and pure water, and the securing of these should be the study of every housewife.

"Nature has most bountifully supplied this city with sunlight, pure air and pure water, and there would seem no excuse for any one's failing to appreciate and appropriate them; yet often, through ignorance or carelessness, these great boons are disregarded and even made ineffective by a want of cleanliness or by the neglect of simple sanitary precautions.

"Those who build residences for themselves should choose a healthy locality, have their house on rising ground, if possible, and see that the plans include windows on all sides to admit nature's great health-giver and purifier—sunlight.

"The next thing to be attended to is the drainage, and no expense ought to be spared to make this as thorough and durable as possible. One whiff of foul air is enough to make some people sick, and sometimes whole families are made seriously ill by a defective drain. A stable should be placed as far away from the house as possible, and be well drained. It is desirable that the kitchen be so arranged that the odors of cooking will not permeate the house. In planning the living rooms they should be made large and light. The bedrooms particularly should be so arranged, an abundance of fresh air being required while we are sleeping, for, when nature if off her guard, insidious enemies more easily assail us.

"Those not fortunate enough to be able to build should select a house very carefully, with an eye to healthiness of neighborhood, proper drainage and free exposure to air and sunlight. Once settled in a home it becomes the duty of the housewife to see that everything pertaining to the health of her family is attended to, not occasionally, but regularly, day by day. Of all the rooms in the house the bedrooms, bathrooms and kitchen require most attention. As soon as the sleeping rooms are vacated in the morning all doors and windows should be thrown wide open and left so as long as possible. The bedding should be spread out to air, if possible outside in the sun. All toilet dishes should be carefully cleansed. At night no soiled clothes should be left in the room, and nothing that would create an odor of any kind, not even an agreeable one; it has often been demonstrated that flowers and perfumes are injurious to sleepers. No caution is necessary as to leaving windows open in summer, but it is just as important to have plenty of fresh air in winter. On the average, every person gives off during a ten hours' sleep six cubic feet of carbonic acid; this requires three thousand cubic feet of fresh air for its dilution, and in the case of sickness a still greater amount is required; so, if we shut out fresh air we condemn ourselves to breathe poisonous gas. Keeping a room well aired in winter does not necessarily imply keeping it too cool for comfort; with careful attention to fires it is easy to secure a constant supply of warm, fresh air.

"Everything in a bathroom should be kept faultlessly clean, and with systematic care this is a simple thing. Sponges, brushes, etc., should be put out to air daily. The bath tub should invariably be well washed after each using, and once a week thoroughly cleansed with sapolio, or some similar preparation. For rust marks the best scouring material is powdered pomice stone and soap. Closets require flushing regularly and scouring every few days with hot suds and sand or wood ashes or ground pomice stone. The three things in the kitchen which most need careful oversight are the sink, the dish rags and the disposal of refuse. The sink should be scoured every day by using a little wood ashes with hot soap suds or with pearline. Every few weeks a little concentrated lye should be allowed to soak slowly through the waste pipe to keep it clear.

"There is 'death in a dish rag' often, and if any housekeeper doubts it let her smell one that has been some time in use. They should be kept well washed and aired, and be renewed very often. No scraps or refuse of any kind should be allowed to accumulate in the kitchen or, indeed, anywhere in the house. All such should be carried out and placed in some covered receptacle until removed by the scavenger. The boxes or cans should be kept as far from the house as possible; if not washable, be sprinkled with sand or earth or lime when emptied, and if not covered, the trash should be carefully sprinkled with earth, sand or dry leaves, to retard putrefaction and lessen the odor.

"The yard should not only be kept free from weeds, but clean and dry. If there are hollows under or by the house where water settles after rain they ought to be filled up. Water grows stagnant very soon in hot weather, and then becomes a source of disease. No refuse of any kind or dirty water should be thrown in the yard. If a house is unfortunately not connected with the sewer care is necessary in throwing out waste water to see that it is scattered as much as possible, and if the sun does not soon dry it up to sprinkle with sand or earth or lime.

"If there is an outer closet not connected with the sewer it requires sprinkling inside with lime, once a week in summer, or to have a bag of copperas (about six ounces) thrown in once a month, or oftener if necessary. If much used such a closet should be emptied once a year, then walls and ceiling whitewashed. A stable requires careful daily attention, especially if near the house. No accumulation of manure, etc., should be permitted. After careful raking and sweeping the floor should be flushed or covered with sawdust. The manure should be removed from the premises.

"It would seem perhaps unnecessary to caution anyone about the use of pure water when we have such a good supply at hand all the time; yet simple precautions are often neglected, especially in winter. Everyone should be aware that water absorbs impurities from the air and is unfit for use after standing for any length of time in a close room and after being in a sick room even for a few moments. If from any cause a fresh supply cannot be obtained the vessel containing it should be kept covered. Another danger from water is using it for drinking or cooking directly from a faucet without allowing it to run for a time. This is a serious mistake, for the water in the pipes absorbs lead, and in greater quantities the purer it is, so that if not allowed to run until pure it will introduce poison into the system.

"Refrigerators need constant care. They require daily airing for about an hour, either at night or very early in the morning. Nothing that is not perfectly fresh should be allowed therein. All liquids (milk especially), butter and all soft things should be kept covered, as they absorb odors. Once a week, during hot weather, every part of the refrigerator, including the tubes, should be thoroughly washed with hot soap suds and rinsed with cold water. Besides the daily care in details, a systematic housecleaning at least once a year is indispensable. This need not be the unpleasant experience it so often is if one room is taken at a time. Carpets and mattings should be taken up and renovated, pictures and all movable things taken out and cleaned, and woodwork washed. For cleaning floors where carpets have lain it is well to have a little carbolic acid in the water to destroy any possible moth eggs, and the water is best used hot. Paint may be washed with ammonia and warm water, or, if much soiled, with washing soda and hot water. Walls, if papered, should receive a thorough dusting with cloths, not feather dusters, for these merely disturb the dust, which settles elsewhere. If the walls are painted or kalsomined it is best to wash them carefully with diluted ammonia, rinsing with clear, cold water.

"All these sanitary suggestions relate to the home where no sickness is, but where that is present special measures are imperatively necessary if the disease is either contagious or infectious. The first precaution is to see that everything used by or for the patient shall not be used by or for any one else. Even the brooms and dusters used in the room should not be used anywhere else; the dusters ought to be burned and the broom soaked in hot water after using, while the sweepings must be immediately carried outside and buried, or completely covered in the trash box. The reason is that disease germs lurk in dust and not in air usually.

"All dishes, cutlery, etc., used should be at once plunged in boiling water. The air in the room should be kept constantly pure. It is a mistaken idea that camphor, cologne and such things do away with the ill effects of bad odors; they merely disguise, without removing them. The only way to keep the room sweet is by keeping everything in it spotlessly clean, and letting in fresh air by doors and windows. This is possible in winter if an extra good fire is kept on during airing time. All soiled linen should be immediately launderied (by itself and not with other clothing), or if this be not possible, put into water containing two per cent. carbolic acid, before being removed from the room.

"When a room is vacated by a consumptive or by a fever patient it is not fit for occupation by anyone else until thoroughly disinfected. The simplest disinfectant is heat, applied either dry or in form of steam or boiling water. All valueless things should be burned, and washable things launderied at once with an extra boiling of at least half an hour. Everything that can be sponged should be treated several times with a three per cent. solution of carbolic acid or a five per cent. solution of chloride of lime or corrosive sublimate—one-half ounce to three gallons water. Goods which cannot be washed may be purified either by dry heat (230 degrees Fahr.) or be subjected to a current of steam (230 degrees Fahr.) for fifteen minutes. All articles carried from the room should first be placed in bags. Then the mattresses, if not removed, should be spread out—open if possible; the carpet, if still in the room, be raised up on chairs and spread out; every crevice in the room be stopped up and fumigation applied. The cheapest and safest fumigator is sulphur, using four pounds to every thousand cubic feet of space. It is better to use equal parts brimstone, broken small, and flowers of sulphur than all of one kind. Place this in an iron vessel (or several vessels if the room is large) over hot coals and leave for at least twelve hours. Boiling water left in the room in open vessels during fumigation makes it more thorough.

"For disinfecting inside closets use a five per cent. solution of chloride of lime or of corrosive sublimate; for outside closets about four pounds of chloride of lime or of copperas.

"The duties of a thorough housewife are, indeed, very arduous, for unending vigilance is demanded of her. Even if she has hired help for all work, constant supervision is required to see that instructions are carried out. But every true woman takes pride in keeping her house and surroundings above reproach, and does not grudge time and effort spent in securing the health and comfort of her family."

